# Prediction of alternatively skipped exons and splicing enhancers from exon junction arrays

**DOI:** 10.1186/1471-2164-9-551

**Published:** 2008-11-20

**Authors:** Katerina Kechris, Yee Hwa Yang, Ru-Fang Yeh

**Affiliations:** 1 Department of Biostatistics and Informatics, Colorado School of Public Health, University of Colorado Denver, 4200 East 9th Avenue, B-119, Denver, CO 80262, USA; 2 School of Mathematics and Statistics, Sydney Bioinformatics, Carslaw Building F07, University of Sydney, NSW 2006, Australia; 3 Department of Epidemiology and Biostatistics, University of California San Francisco, Box 0560, San Francisco, CA 94143, USA

## Abstract

**Background:**

Alternative splicing of exons in a pre-mRNA transcript is an important mechanism which contributes to protein diversity in human. Arrays for detecting alternative splicing are available using several different probe designs, including those based on exon-junctions. In this work, we introduce a new method for predicting alternatively skipped exons from exon-junction arrays. Predictions based on our method are compared against controls and their sequences are analyzed to identify motifs important for regulating alternative splicing.

**Results:**

Our comparison of several alternative methods shows that an exon-skipping score based on neighboring junctions best discriminates between positive and negative controls. Sequence analysis of our predicted exons confirms the presence of known splicing regulatory sequences. In addition, we also derive a set of development-related alternatively spliced genes based on fetal versus adult tissue comparisons and find that our predictions are consistent with their functional annotations. *Ab initio *motif finding algorithms are applied to identify several motifs that may be relevant for splicing during development.

**Conclusion:**

This work describes a new method for analyzing exon-junction arrays, identifies sequence motifs that are specific for alternative and constitutive splicing and suggests a role for several known splicing factors and their motifs in developmental regulation.

## Background

Eukaryotic gene expression is controlled by a series of biological events involving various interactions among proteins, DNA and RNA that are subject to complex regulation. One of the essential processes is pre-mRNA splicing, in which the spliceosome complex recognizes splice sites (SS) in the precursors of mRNA following transcription, removes noncoding introns and joins neighboring exons to form mature messenger RNA that can be further translated into protein sequence. Both 5' splice sites (marking exon-intron boundaries, or *donor *sites) and 3' splice sites (marking intron-exon boundaries, or *acceptor *sites) consist of short basal signals well conserved from yeast to human, but contain insufficient information to accurately identify exon-intron boundaries in vertebrates [1]. Consequently, there are numerous cryptic splices embedded throughout the pre-mRNA transcript. Variations in the splice site detection process can create multiple transcript variants for a gene and is referred to as alternative splicing. Alternative splicing events may include the skipping or retention of entire exons, intron retention or alternative usage of 3' or 5' splice sites. These changes often lead to modifications in the encoded proteins and have been shown to play a critical role in development and disease [2-4]. Alternative splicing also provides an efficient means to expand protein diversity from the limited gene pool. It is estimated that 50–80% of the approximately 25,000 human protein-coding genes are subject to alternative splicing [5-7] underscoring the importance of splicing regulation.

The correct recognition of splice sites is facilitated by various protein-protein and protein-RNA interactions. In addition to exon-bridging or intron-bridging interactions of splice sites, there are many non-splice-site sequences in exons and adjacent introns, termed enhancers or silencers, that stimulate or repress the splicing of constitutive or alternative exons. Both enhancers and silencers have been identified through *in vitro *and *in vivo *methods [8-11]. Large-scale identification of these sequence elements are based on computational methods which analyze multiple sequences simultaneously. Some authors have focused on constitutively spliced exons [12-15], while others have focused on identifying the sequence signals that discriminate between constitutively and alternatively spliced exons [16-21]. More recent methods now take advantage of the availability of genomes from related species to make predictions based on evolutionarily conserved sequences [22-25]. Although motif search methods such as [26] identify sequences that are similar to previously determined enhancers and silencers, most computational approaches require as input a large collection of alternatively spliced exons.

The most widely used method to identify alternative splice variants is based on aligning EST and mRNA sequences to the genome [27-29]. Application of this method has provided estimates for the percentage of human genes with multiple splice variants between 40% and 60% [27-29]. However, EST-based methods are often biased towards the transcript ends, prone to sequencing errors, biased towards more highly expressed genes, limited by the availability of clone libraries for particular cell and tissue types and not uniformly annotated. The recent adaptations of microarray technology in the form of splice arrays are now providing new directions for detecting alternative splicing. DNA microarrays were originally developed to measure expression levels for a large number of genes simultaneously. Arrays for detecting alternative splicing (splice arrays) contain probes specifically designed to investigate splice events. They have been used to explore splicing in yeast.[30,31] and in mammalian cells (*e.g.*, [6,32-34]). Several different designs reviewed in [35] have been developed including tilling arrays, arrays with specific probes that distinguish between known splice forms [32,36], arrays based on exon probes [37,38], exon-junction probes [6,39] or both [32,36,40,41]. Most of the data analysis methods for splice arrays and levels of alternative splice inference depend on the array design (see review in [35]), and the design of these arrays typically rely heavily on known or predicted annotation of gene structures. In summary, splice arrays have been shown to complement the EST-based approaches by identifying a large number of novel alternative splices with no previous EST support, despite a moderate validation rate of ~50% by RT-PCR [6,42,43].

In this paper, we propose a new statistical approach for identifying alternatively spliced exons from exon-junction arrays, and predict a large set of alternatively skipped exons. This is achieved using the most comprehensive exon-junction array analysis to date [6], which monitored all exon junctions of over 10,000 RefSeq genes in 52 tissues samples. Our approach consists of a novel exon-skipping score that serves as a quantitative measure of evidence for alternatively skipped exons. By applying kmer-contrast and regression-based sequence analysis methods to the top ~8400 exons according to our score, we are able to recover several known splicing enhancers and identify additional novel candidate splicing regulatory motifs associated with skipped exons. Finally, we also identify a set of development-related alternative splices and their associated enhancers using a tissue-pair analysis followed by *de novo *motif finding algorithms. Enrichment analysis of gene ontology annotation supports the functional roles of the predicted development-related alternative splices and suggests a new scheme for identification of process-specific alternative splicing.

## Results and discussion

### Analysis of exon-junction array

The pre-processed array data described in [6] were obtained from the NCBI Gene Expression Omnibus (GEO) database with accession number GSE740. The dataset contains the average background subtracted intensity *y*_*i*, *j*, *k *_from two dye-swapped arrays for each exon-junction probe *j *of gene *k *in tissue *i*, where *i *is one of the 52 tissues surveyed and *j *= 1, ..., *n*_*k *_for gene *k*, which has *n*_*k *_probes bridging *n*_*k*_*+1 *exons. Note that intensity patterns of exon-junction (EJ) probes alone cannot distinguish between intron retention or alternative 5' or 3' splice site usage, and hence we only focus on the effective detection of exon skipping in the 8618 genes containing 5 exons or more (*n*_*k *_≥ 4 on the array). For each gene, we fit a linear model with terms for probe and tissue effects. Because of the large number of effects that need to be estimated, we must restrict the analysis to genes where there are enough data points. We found that a minimum of 5 exons was an adequate cutoff that avoids over-fitting in our statistical analysis but does not filter too many genes. For each gene, 5 exons corresponds to 4 exon-junctions (4 data points per tissue) and consequently 3 adjacent exon-junction pairs.

If we treat exon inclusion as a simplified binary "on" or "off" event in each tissue, then conceptually, there are three main signal sources, other than noise, for the observed EJ probe intensity *y*_*i*, *j*, *k*_: (i) probe-specific effect describing sequence binding affinity, (ii) tissue-specific effect resulting from differential expression, and (iii) alternative splice (AS) effect determined by exon inclusion or alternative splice site selection. A simple approach to recover the variance components of individual signal contribution is to use linear models on the properly transformed data (EJ probe intensities), essentially assuming that the transformed intensity is the sum of the probe, tissue, AS effect and white noise. A skipped exon will result in the two spanning EJ probes to be switched "off". This supports an exon-skipping score that measures the magnitude and concordance of AS effects in adjacent EJ probes spanning the same exon. Identifying exons that are alternatively skipped in some of the tissues surveyed is then equivalent to the detection of scores that deviate across tissues. In summary, our data analysis method can be described as a three-step procedure:

1. Estimate the variance stabilizing transformation [44] for the EJ probe intensities to satisfy the constant variance assumption for linear models.

2. Fit a linear model on the transformed data to estimate probe and tissue effects. The residuals of the fit *r*_*i*, *j*, *k *_represent AS effects adjusted for probe- and tissue-specific effects.

3. Summarize exon-skipping events at the exon and gene level by defining an exon-skipping score based on the residuals *r*_*i*, *j*, *k *_and test for large deviations of this score across tissues.

Note that [6], among others (*e.g.*, [45]), also used a linear model on log-transformed (step 1) intensities but estimated linear model parameters by medians (step 2), followed by *ad hoc *thresholding of the residuals *r*_*i,j,k *_to define AS scores (step 3). We considered several alternatives to this approach. First, the original method of [6] only examined individual exon-junctions by thresholding the residuals into four categories from 0,1,2 and 3. The largest residuals, those given a score of 3, were predicted as splicing events. For each junction, the authors then tallied the number of tissues where the residuals had a score of 3. There appeared to be no systematic evaluation of neighboring pairs of exon-junctions in this scoring procedure. Therefore, as an alternative, we calculated the novel statistic of residual products so that both flanking exon-junctions for an exon contribute to its score. Second, our preliminary analysis (data not shown) indicated that the logarithm function used by [6] to stabilize the variance may be insufficient. Therefore, we also tried alternative transformations. Third, we used statistical significance cutoffs to threshold the residuals (or residuals products).

In particular, at each stage of the three-step procedure, we considered several alternative methods for the same objective: applying the logarithm or arcsinh-based variance stabilizing transformation (VSN) function for stabilizing variances in step 1; using the standard least square fit (mean) or robust fit (median or median polish) for linear model parameter estimation in step 2; and measuring deviations from normality of residuals in step 3 using the non-parametric Kolmogorov-Smirnov (KS) test, a weighed non-parametric Kolmogorov-Smirnov test (WKS), parametric Wilk-Shapiro test (WS) [46] or simple thresholding of the coefficient of variation (CV, standard deviation normalized by the mean) on the residuals or residual products (see Methods). We viewed the selection of these options as an optimization problem, and determined the best procedure in terms of prediction accuracy using control data. We used two sets of alternatively and constitutively spliced exons curated from independent sources as controls (see Methods). The positive control (AS) consists of 164 genes supported by EST data. The negative control (CS) consists of 282 genes selected from the analysis of an Affymetrix exon array experiment that show constitutive splicing across a panel of 11 tissues. These two examples represent "approximate" controls because they rely on available ESTs or other data on selected tissues. However, we expect that they will be dominated by the desired events.

**Figure 1:**
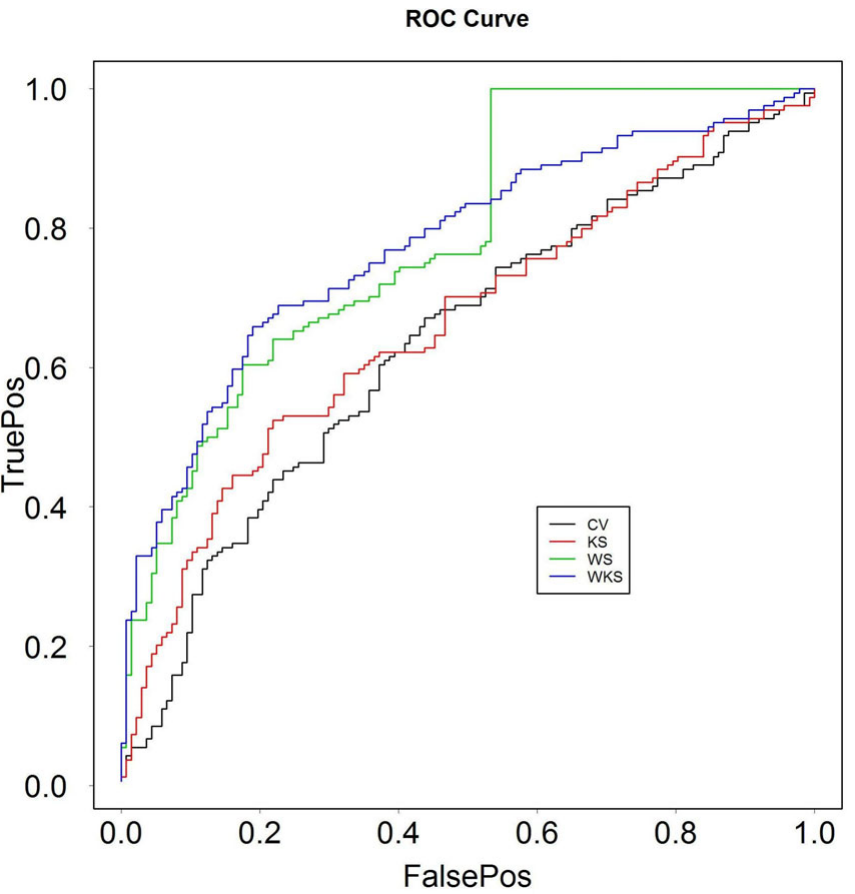
**ROC curve comparing step 3 options based on control data sets**. The x-axis is the percentage of false positives (FalsePos) and the y-axis is the percentage of true positives (TruePos). The step 3 options described in the text are CV (thresholding of the coefficient of variation), KS (Kolmogorov-Smirnov test), WS (Wilk-Shapiro test) and WKS (weighted Kolmogorov-Smirnov test).

**Figure 2:**
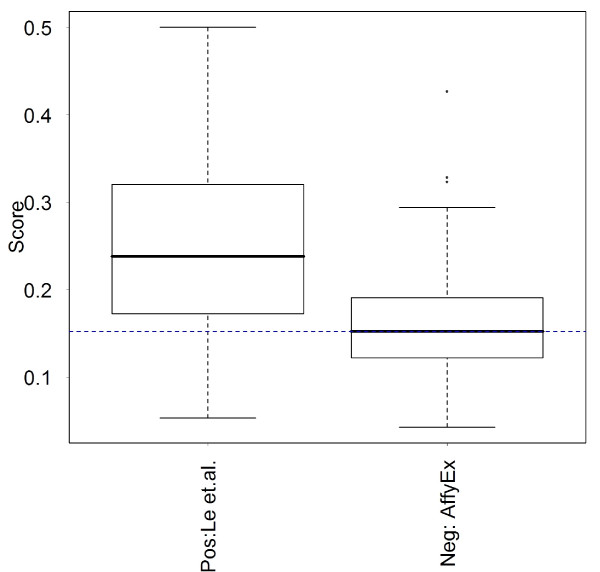
**Boxplot of WKS-statistic (y-axis) at the gene level for each of the control sets**. The positive control set is labeled "Pos: Le et al." and the negative control set is labeled "Neg:AffyEx".

Using the control data sets and receiver operating characteristic (ROC) curve analysis, we found that the VSN transformation performs better than log-transformation as expected (data not shown), and that the fitting procedures in Step 2 were indistinguishable in terms of prediction errors (data not shown). Figure 1 shows the improvement of our procedure (VSN, least square fit, WKS) over our best reproduction of the method described in [6] using exon-level summaries (CV score, see Methods). Note that the AS calls in the previous work [6] were calculated from consistent AS scores from individual arrays which were not publicly available (only the average intensity of dye-swapped replicates were released). Therefore, we could not reproduce previous results in [6] but believe that the CV score is similar in spirit to that approach. Figure 2 shows the distribution of the WKS-statistic at the gene level for the two control sets. Overlaps in the distribution between the positives and negative may be due to the caveats regarding the control sets discussed above. Nevertheless, the values of the WKS-statistic are generally higher for the positive controls versus the negative controls.

To further validate these results, we examined the WKS scores on another set of positive controls; the positive RT-PCR results in [6] and the 2656 RefSeq entries on the array with "cassetteExon" events from the UCSC "knownAlt" table hg18 annotation [47]. Although the RT-PCR set also contain genes with alternative splice site selections and intron insertions, the two new combined positive controls had WKS-statistics significantly greater than the negative control (Mann-Whitney test p-value = 4.9 × 10^-4^). Several of the highest scoring examples using the WKS method, such as UBA3 (ubiquitin-activating enzyme 3 isoform 1), MAPKAP1 (mitogen-activated protein kinase associated) and PPM1B (protein phosphatase 1B isoform 1) were identified as "cassetteExon" gene entries from UCSC but were low scoring using the CV method, which only uses data from a single exon-junction. This illustrates that the combined product score based on neighboring exon-junctions can predict known examples which are missed by single junction methods. In summary, given the original linear model in [6], we derived a new exon-skipping score that 1) is based on an alternative transformation which improves the correction for non-constant variance and 2) draws on information from both splice junctions of an exon using the product residuals.

Using a score cutoff that balances the rate of true positive and false positives in our original two controls, we identified a set of alternatively spliced genes and corresponding exons (see Methods). We then determined the lowest ranking genes and exons according to our score to obtain an equivalently sized set of constitutive spliced exons. In total we predicted 8433 alternatively skipped exons and 8113 constitutive exons. An analysis of the Gene Ontology (GO) terms [48] for our predicted alternatively spliced genes (see Methods) using GOstat [49] showed enrichment in 114 GO terms. More than half of these GO terms are related to metabolism, cell death, regulation, transcription, splicing and protein targeting and localization (See Additional file 1, Table S1), which are categories consistent with prior studies [19,29,45].

### Sequences associated with alternatively splicing

To identify sequence motifs associated with the regulation of alternatively skipped exons, we adapted the contrast-kmer-based RESCUE-ESE algorithm for splicing [12] and the regression-based REDUCE program for transcription factor binding sites [50]. The RESCUE-ESE algorithm accounts for the fact that splicing enhancers compensate for weak splice site signals, and finds candidate exonic splicing enhancers (ESE) for constitutive splicing from statistically over-represented hexamers in exons versus introns and associated with exons defined by weak splice sites versus strong splice sites. REDUCE enumerates all possible kmers in the promoters up to a specified length and uses a linear regression model to find kmers that show significant correlation with gene expression levels from a single microarray experiment. To identify putative splicing regulatory elements associated with alternative splicing, we used different contrast sets for RESCUE-ESE and employed our exon-level statistic for REDUCE on exons and their flanking intron sequences.

### Exonic splicing enhancers (ESE) for alternatively and constitutively spliced exons

**Figure 3:**
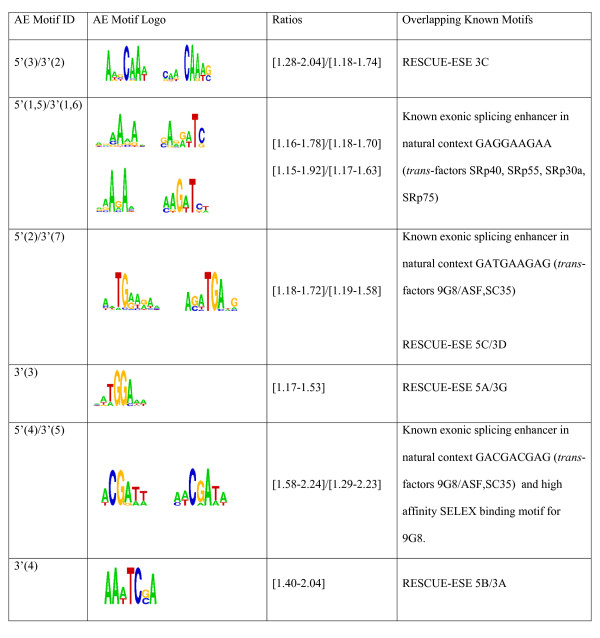
**Predicted non-redundant 3' and 5'SS associated AE motifs and their overlapping known motifs**. Predicted AE motifs IDs and motifs are listed in columns 1 and 2. The motifs are displayed graphically as logos in the order of the IDs in column 1. The Motif ID includes a label for whether it was discovered relative to the 5' or 3' splice site and a numeric identifier for the motif in parenthesis. For all the kmers that define each motif in Column 2, Column 3 lists the range of ratios of the occurrence of these kmers in alternative exons versus constitutive exons. See Additional file 1, Tables S2 and S3 for the list of all motifs, their kmers and their numeric identifier. RESCUE-ESE and known enhancers (column 4) are taken from the survey in [12].

A direct contrast of alternatively skipped exons (AE) versus constitutively spliced exons (CE) in addition to differences in exons versus flanking introns gave 6 motifs associated with the 5' splice site of AE (204 hexamers in Additional file 1, Table S2) and 7 motifs for the 3' splice site of AE (192 hexamers in Additional file 1, Table S3), among which 5 motifs are in common. Several of the motifs overlap known ESE motifs from deletion experiments, functional SELEX or SR protein binding sequences (see survey in [12]), such as the purine-rich AAGA for SRp40, SRp55, SRp30a and SRp75, ACGA and TGAAG for 9G8, SC35 and ASF, and consensus GAAG for Tra2β [51] and motif variants for ASF/SF2. Three of the 8 nonredundant motifs (Figure 3) also match RESCUE-ESE predicted and experimentally verified motifs for constitutive splicing from [12], indicating that similar ESEs and splicing factors are involved in both alternative and constitutive splicing. For the kmers that define the predicted motifs, there are ~1.1 to 2.2 times more kmer counts in alternative exons versus constitutive exons (Figure 3).

**Figure 4:**
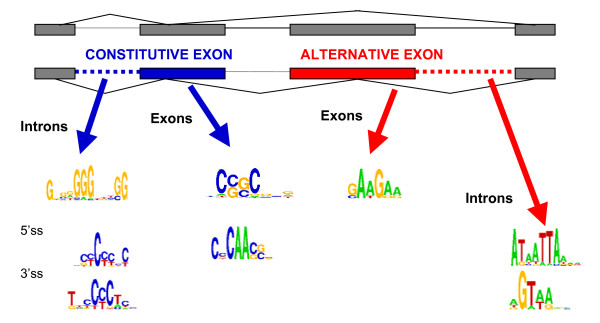
**Summary of motifs found in alternative and constitutive exons and flanking introns**. Two alternative splice forms are indicated at the top. An alternatively spliced exon for the splice forms is labeled in red while a constitutively spliced exon is labeled in blue. The flanking intron for the alternative and constitutive exon is indicated by the dashed line. The arrows point to representative motifs associated with each of the sequences. Motifs found with respect to the 5' or 3'SS introns flanking constitutive exons are labeled separately.

Interestingly, when the hexamers were subjected to the additional contrast of splice site strength association (weak splice site exons versus strong splice site exons) as in the RESCUE-ESE analysis for constitutive splicing, the number of significant kmers reduced drastically. The only significant AE motif associated with weak splice sites is the well known motif GAAGA from 13 and 6 non-redundant hexamers in the 5' and 3' analysis respectively (Figure 4 and Additional file 1, Table S4). Two other AE-associated ESE motifs, an A-rich and a TGGA motif, are linked to strong splice sites. These results suggest that the GAAGA motif and its associated splicing factors (9G8, ASF, SC35, SRp40, SRp55, Rp30A, SRp75 all have been implicated) may be critical for regulating alternative exons in the presence of very weak splices. This result may be due to the observation that splice sites involved in alternatively spliced exons tend to be weaker overall [25] and therefore, splice site strength dependency will only pick up the strongest enhancers. Using position-specific scoring matrices to evaluate splice site strength, we did not observe a bias in weaker splice sites in the entire group of AE versus CE. However, correcting for overall gene effects, the splice site scores in the AE were relatively smaller than the scores in the CE (see Additional file 2).

We also focused the contrast analysis on constitutively spliced exons to select for ESEs relevant for constitutive splicing using CE versus AE and exons versus introns comparisons and found 23 motifs for the 5' analysis and 12 motifs for the 3' analysis. The majority of motifs detected are CG-rich with diverse consensus patterns, most likely reflecting the coding bias of CE (Figure 4 and Additional file 1, Tables S5 and S6). Besides the CG-rich motifs, 6 of the 23 non-redundant motifs found also overlap known motifs or natural occurring enhancers, including CA-rich motifs (CAAC, CCAC) for splicing factor YB1, TGCCGTT for SC35 and functional SELEX motifs for Schaal-II-D (TCTCC) [12]. The resulting motifs were similar when we also restricted the comparison to weak splice sites. Finally, no motifs were found to be associated with strong splice sites in support of CE, consistent with the notion that splicing enhancers are required only when splice sites do not contain enough information in order to facilitate unambiguous exon recognition.

Alternatively, we applied the REDUCE software to correlate sequence features with the exon-skipping score (see Methods). Table 1 shows the most significant kmers (p-value < .01, Bonferroni corrected). Kmers positively correlated with the exon-level skipping score are associated with alternatively spliced exons, while negatively correlated kmers are associated with constitutively spliced exons. Note that this regression-based approach is a natural extension of contrast analysis of AE versus CE, and aims to evaluate the sequence association at the genome scale without the need to dichotomize scores. The REDUCE kmers were consistent with the above RESCUE-ESE analysis; the top negatively correlated kmers for CE are primarily G/C rich with a CACC-containing motif (tCACCg), and the AAGAA motif was found to be the top candidate for AE.

**Table 1:** Significant kmers found by the REDUCE algorithm.

Kmer	Correlation	P-value
cctgg	-0.0451	3.6e-36
aagaa	0.0310	1.8e-16
cgtgg	-0.0233	1.1e-08
gtttt	0.0195	1.0e-05
gggaga	-0.0199	2.1e-05
tatgg	0.0169	7.0e-04
tcaccg	-0.0178	7.0e-04
tcaac	-0.0161	2.2e-03
gaagat	0.0170	2.4e-03
tggagg	-0.0168	3.3e-03
gccgg	-0.0151	8.1e-03

The kmer, the correlation between the kmer counts and exon-skipping score for each exon, and the p-value for the correlation are listed in columns 1, 2 and 3 respectively.

We applied similar contrast analysis to identify intronic motifs associated with AE by selecting for kmers over-represented in introns flanking AE versus CE-associated introns and in introns versus exons. We found A/T rich motifs associated in introns flanking AE and pyrimidine tract-like motifs and G-triplets in introns flanking CE (see Figure 4, Additional file 2). Randomization runs were performed on the exon data to assess the frequency of observing the predicted kmers in random data. The results indicate that we are observing more predicted kmers than expected by chance (see Additional file 2).

### Development-related alternative splices derived from tissue-pair analysis

**Figure 5:**
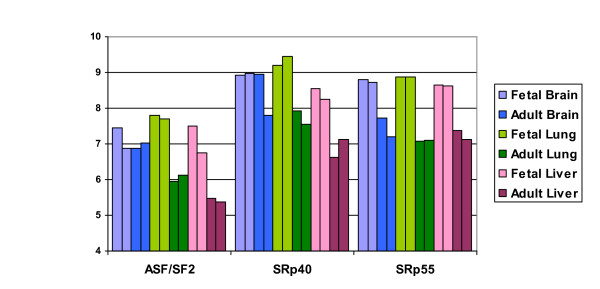
**Differences in log-intensities (y-axis) between fetal and adult tissues for three SR proteins**. There are two replicates for each fetal and adult tissue, which are labeled with the same color shade. Tissues (brain, lung and liver) are labeled in different colors.

To investigate whether *trans*-acting elements (*i.e.*, protein-RNA binding proteins) are relevant for development, we analyzed a comprehensive gene expression data set from the Gene Expression Atlas [52], which contains results from similar tissues lines as the exon-junction array. The CEL files of the Gene Expression Atlas data were provided by the authors and the data were preprocessed using robust multi-array analysis (RMA) proposed by [53]. We extracted genes encoding the family of serine/arginine (SR) proteins, which contain both RNA-binding and protein domains and are known to facilitate the assembly of the spliceosome by binding to ESEs [54]. Figure S1 in Additional file 3 shows the processed log-intensity values in adult and fetal lung, brain, and liver tissues for SR proteins listed in [20]. In particular, SRp55, SRp40 and ASF/SF2 (Figure 5) clearly show reduced expression in the adult tissues compared to the fetal tissues in at least two of the three examined tissue types.

These results imply that some SR proteins may be involved in a general developmental response. To explore this hypothesis, we further investigated the exon-junction array, which has extensive results from different tissues (*e.g.*, liver), developmental stages (*e.g.*, fetal) and disease cell lines (*e.g.*, lung carcinoma). Therefore, it is possible to identify AS involved in specific biological process by examining splice patterns between tissue pairs. For instance, genes that show different splicing profiles in fetal versus adult brain tissues may be indicative of functional roles in the regulation of brain development through splicing control. By comparing fetal versus adult tissues for several different organs, we adapted our gene-level exon-skipping score to obtain alternatively spliced genes that are associated with development.

Within one gene, for each tissue *i *we have the product score *r*_*ij *_across exons *j*. We are interested in genes where there are large differences in *r*_*ij *_for pairs of tissues (*e.g.*, fetal lung versus adult lung). Define *d*(*i*, *i*') = *r*_*ij *_- *r*_*i*'*j *_as the difference between two tissues, *i *and *i'*, in exon *j*. Extreme values (both positive and negative) for *d *indicate differences in exon-skipping levels between the tissues for that exon. We calculated a gene-level tissue pair difference score as the KS-statistic of *d *across all exons relative to a simulated null model assuming normally distributed residuals *r*_*ij *_to gauge the differences in exon-skipping patterns between two tissues. Genes with Bonferroni-adjusted p-value < 0.05 were selected as candidate genes for development-related alternative exon-skipping in brain, liver, and lung tissues (Table 2). Many of our predicted genes are known to have multiple splice variants which are regulated during development, including neurofibromin 1 (NF1) in the brain for the RAS signal transduction pathway [55,56], L1 cell adhesion molecule (L1CAM) for nervous system development [57] and ion transport pump gene ATPase isoform 4 (PMCA4) [58] and fibroblast growth factor receptor 1 (FGFR1) for cell division in brain development [59,60].

**Table 2:** Development-related alternatively spliced genes.

	Lung	Brain	Liver
Significant genes	395	631	482

Genes with PubMed tissue keyword	27 (7%)	127 (20%)	52 (11%)
% of all genes with keyword	5%	11%	7%
p-value	3.57e-02	1.04e-12	9.62e-04

Genes with "splicing" keywords	92 (23%)	135 (21%)	93 (19%)
% of all genes with keywords	12%	12%	12%
p-value	5.46e-11	1.26e-12	8.01e-07

Genes with PubMed tissue, "development" or "splicing" keywords	135 (34%)	258 (41%)	160 (33%)
% of all genes with keywords	23%	26%	25%
p-value	3.14e-07	4.57e-16	2.21e-05

A keyword hit is defined as a gene that has at least 2 abstracts with the keyword. The numbers in parentheses indicate the percentage of genes with a keyword hit within that gene set. Tissue keyword is the tissue name.

To systematically assess the functional validity of our predictions, we analyzed the functional annotation of the predicted genes in the literature. Tissue-specific keywords were searched in the abstracts listed in the PubMed entries for each gene (see Methods). For example, for our lung-developmental genes (column 1 in Table 2), we searched for the keyword "lung" in each abstract, and a p-value was derived for tissue-specific keyword enrichment compared to all genes on the exon-junction arrays using Fisher's exact test (Table 2). We also specifically searched for a splicing-related keyword to check that the tissue annotations are not only due to transcription related information. In summary, all our predicted tissue-specific gene sets showed statistically significant (p < .05) enrichment for the respective tissue-specific keyword, splicing-related keyword and for a combination of tissue-, splicing- and development-related keywords. A separate analysis of the GO terms in the 127 predicted brain-development associated genes with the "brain" keyword in PubMed showed functional enrichment in 26 GO terms using GOstat (Benjamini-corrected false discovery rate < 0.01). The most common GO terms (11 out of 26) were those with functions related to signaling and ion transport (Additional file 1, Table S12), which are relevant for synaptic transmission and neurogenesis. In summary, the two annotation analyses collectively support the validity of our predicted genes.

**Figure 6:**
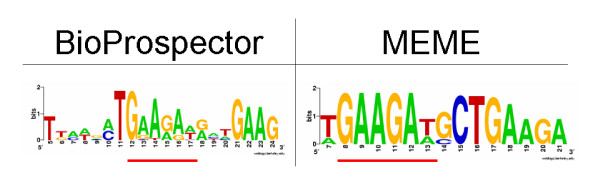
**Motifs found in development-related exons**. Results are based on two motif-finding algorithms, BioProspector and MEME (see Methods). The common conserved sequence is underlined.

To identify potential cis-regulatory elements involved in splicing regulation for development, we applied *de novo *motif finding algorithms MEME [61] and BioProspector [62]. To keep the number of exons manageable for these algorithms, only genes predicted to undergo development-related AS for all three tissues and had a "splicing" or "development" keyword hit from the PubMed abstract search were considered. Applying the motif algorithms to alternatively spliced exons within these genes (see Methods and Additional file 1, Table S13), we repeatedly found a "GAAGAA" motif (Figure 6), which is very similar to the AE motif retrieved by our previous analyses and suggests that this motif and its binding factors are also involved in the developmental regulation of splicing. Furthermore, since we previously found that this motif was more strongly associated with weak splice sites rather than strong splice sites, these results also suggest that the mode of regulation for splicing during development may be due to the combination of a weak splice site and the presence of this enhancer. However, the appearance of the "GAAGAA" motif in exons predicted to undergo development-related AS may just be an artifact of this motif appearing in AS exons in general. To investigate this scenario, we checked whether the "GAAGAA" motif appeared in the development-related AS exons more frequently than all AS exons (AE from above). We counted the occurrence of the motif in both sets of exons and found that indeed the occurrence of the motif was significantly higher than expected in the development-related AS exons (p-value = .02, binomial test) based on the frequency of the motif in general AS exons.

Experimentally verified binding sites in their natural context and from SELEX experiments are listed in Additional file 1, Table S14 for the three SR proteins that we identified based on the expression data as possibly involved in general developmental roles (SRp55, SRp40 and ASF/SF2). Most of these sequence elements contain the "GAA" sequence and are similar to the motifs discovered by our motif search in the development set of AS exons (Figure 5). The known binding sites, expression data, annotation results, and motif analysis provide consistent evidence supporting the hypothesis that these proteins may be involved in the regulation of alternative splicing during development.

## Conclusion

We have developed a novel approach for the analysis of exon-junction arrays that will specifically search for exon-skipping alternative splice events. Our proposed score evaluates the product of expression levels across exon-junctions to more accurately reflect exon-skipping. Our score also accounts for overall expression levels for a gene and uses an improved variance stabilizing correction. We have shown that the combination of these approaches improves the discrimination of positive and negative control sets determined from independent data sources. We could not, however, directly compare our method with existing methods in [6] because that analysis was based on individual array replicates, which we could not access. Nevertheless, utilizing another source of external validation, we demonstrate that the annotations for our alternatively spliced gene predictions were consistent with previous literature. There are several other exon-junction studies, but our method was not applicable because they were either disease specific or a detailed examination of a relatively smaller number of genes [39,63,64].

Following the array analysis, we examined our sequence predictions for dual purposes; to validate our analysis of the exon-junction array and to predict novel splicing enhancers and silencers. The results from the sequence analysis provide a further source of validation for the quality of our alternative and constitutive exon predictions. Using randomization trials, we found that our predictions were enriched in sequences that were not contained in random sets of sequences. We also identified several sequence signals that are consistent with the experimental literature (*e.g.*, GGG triplets and pyrimidine-rich motifs in introns) and identified several motifs that are highly specific. For example, the known exonic sequence enhancer CACC was discovered in the constitutive exons but not in the alternative exons. Another known enhancer, GAAGAA, appears to be more specific to alternative exons than constitutive exons. Furthermore, we found that the occurrence of GAAGAA is biased towards exons with weak splice sites, while the other identified motifs associated with alternatively skipped exons do not have splice strength specificity. The sequence analysis also shows that, although originally developed for gene expression data [50], correlation-based methods utilizing whole genome data like REDUCE are applicable to splice arrays and corroborate the kmer enrichment analysis without relying on pre-determined cutoffs.

By our definition, the alternative exons show patterns of exon skipping among different tissues. The presence of known exonic enhancers in these sequences supports the hypothesis that depending on tissue-specific expression, the corresponding binding factors are enhancing the splicing of these exons, which would otherwise be skipped. An alternative hypothesis, not supported by these results, but also discussed in the literature [10], is that silencer sequences in the exon or flanking introns prevent proper splicing and are responsible for exon-skipping. These hypotheses are not mutually exclusive because both enhancer and silencer sequences may function cooperatively in splicing regulation. Furthermore, some sequence elements have been shown to act as both enhancers and silencers [65].

A useful feature of the Rosetta array is that both fetal and adult samples were included for three different tissues. We used this to develop a method for predicting genes with pairwise tissue differences in exon-skipping and applied our procedure to explore both the *cis *and *trans*-regulation of splicing during development. Our method for pairwise tissue analysis is not limited to the developmental comparison but can be extended to other tissues or cell lines (*e.g.*, cancer versus normal cell lines). We made gene predictions for three tissue types (lung, brain and liver) but only looked at the intersection to focus on general developmental alternative splicing, which was motivated by observed expression changes in specific SR proteins. Our final predictions for development-related alternative splicing were consistent with functional annotation and literature searches. In our sequence analysis of the development-related predictions, we found that a form of the GAAGAA motif may also have a role in alternative splicing during development. Furthermore, the changes in gene expression between fetal and adult tissues for several SR proteins that bind to this motif provide further evidence for their roles in developmental regulation.

Because of the array design, our work focuses on the detection of exon-skipping. However, these data can also be used to predict and analyze other types of alternative splice events. In particular, if alternative splice site selection or intron retention occurs, we would observe variation in a single exon-junction across tissues, but not necessarily in consecutive junctions, as observed with exon-skipping. Therefore, a product summarization step would not be necessary, but we could adapt our procedure in other ways to predict alternative splice selection and intron retention. We will, however, not be able to discriminate between these other types of splice events because of the nature of the array design.

A recent direction taken by several groups is to use sequence conservation across multiple species to aid in the search for enhancers and silencers [22-25,66,67]. Comparative approaches have also been used to examine the conservation of alternative splice events by comparing genomic or EST data from multiple species [29,68-71]. As more splice array data become available from different organisms and tissues, there may also be opportunities to explore the conservation of splicing events from meta-analysis of splice arrays, without relying on ESTs from orthologous genes that are often limited across species.

## Methods

### Exon-skipping scores

Within each gene, the alternative splice (AS) effect for every probe *j *and tissue *i *is estimated by the residual *r*_*i*, *j*, *k *_from a linear model fit. To identify whether exon-skipping has occurred in some tissues, we considered four exon-skipping scores that measure the deviations of the residuals across tissues. All calculations described in this and all other Methods sections are performed in the software R  unless otherwise noted.

One score is based on the two-sample Kolmogorov-Smirnov (KS) test statistic of the residual products of adjacent exon-junction (EJ) probes spanning exons *j, p*_*i*, *j*, *k *_= *r*_*i*, *j*, *k*_*r*_*i*, *j* +1, *k*_. For the exon-level score, residual products across tissues *i *for exon *j *are examined, and for the gene-level score, the residual products across tissues *i *and exons *j *for gene *k *are examined. To obtain p-values based on the two-sample KS test, we compared the observed residual products with randomly sampled residual products from the null hypothesis that that they are a product of normally distributed random variables with mean 0, *r*_*i*, *j*, *k *_~*N*(0, σ^2^), where σ is sampled from the empirical distribution for all genes.

Exploratory data analysis showed greater variability for genes with low overall expression signal and thus we considered a more appropriate exon-skipping score, the two-sample weighted Kolmogorov-Smirnov (WKS = *w*_*k*_*KS*_*k*_) test statistic. The linear weight function is constructed to adjust for the overall expression signal strength and results in increasing the weight of more highly expressed genes. It is defined as $w_{k}=a_{k}/(\underset{k}{\max}a_{k}-\underset{k}{\min}a_{k})$ with $a_{k}=\underset{tissue\;i}{\max}\underset{exon\;j}{\min}y_{i,j,k}$, where *y*_*i*, *j*, *k *_is the average background subtracted intensity. To obtain p-values, observed values of WKS were compared with randomly sampled WKS statistics based on the product of randomly sampled weights, from a uniform distribution within the empirical range of calculated weights for all genes, and randomly sampled KS statistics, sampled from the theoretical distribution of the KS statistic.

Another score is based on the coefficient of variation (CV), a statistic that measures large variations and is defined as the standard deviation divided by the mean. For exon-level score, we calculated the CV of the residual products across tissues, $\underset{tissue\;i}{CV}(p_{i,j,k)}$ and for the gene-level score, we calculated the standard deviation across exons within the gene, $\underset{exon\;j}{sd}[\underset{tissue\;i}{CV}(p_{i,j,k})]$. This method is closest to the Rosetta approach [6] which relies on a statistic similar to the coefficient of variation.

Finally, we considered a score based on a direct test for normality using the Wilk-Shapiro test (WS) [46]. In this case we did not use the product summarization, but tested the following hypothesis using the WS test: *r*_*i*, *j*, *k *_~*X *where *X *is distributed as *N*(0, σ^2^). As before, the tests are performed across exons or genes to obtain the exon- or gene-level scores respectively.

### Controls

A collection of sample Affymetrix Human Exon 1.0 ST array data (from [72]) was used to generate an independent set of negative controls. These arrays represent a collection of 11 tissues with 3 replicates each. All of the 11 tissues were included in the Rosetta data set [6] except breast. Identifying gene sets from these arrays that demonstrate no changes across all 11 tissues provide an independent source of experimental evidence for constitutive exons. The data are background-corrected, quantile-normalized and summarized at the exon level using the Robust Multi-Array Average (RMA) method [73] to generate a logarithm base 2 RMA value, *x*_*i*, *j*, *k*_, for each exon probe set. To identify genes that are likely to be constitutively spliced, we rank genes according to their expression variability across the 11 tissues and across corresponding core exon probesets, $affy_{k}=\underset{exon\;j}{range}\underset{tissue\;i}{range}x_{i,j,k}$. The top 5% least variable RefSeq genes that are both present in the Affymetrix exon arrays and Rosetta exon-junction arrays (n = 282) were selected as our negative controls.

The positive control (AS) consists of 164 EST-supported alternatively spliced genes that were placed on the arrays in Le et al., 2004 [42].

### Constitutive and alternative exons

To determine a set of alternatively spliced genes, we used a cutoff for the gene-level WKS-statistic that produced a true and false positive rate of ~70% and ~25% respectively on the controls. This cutoff resulted in 3912 predicted alternatively spliced genes. Of the exons in these genes, we then extracted exons in the 75^th ^percentile according to their exon-level KS-statistic, which resulted in 9178 predicted exons. The KS-statistic is used for the exon-level score because the WKS-statistic has a gene-level correction for expression variability that is the same for all exons in a gene. Using the UCSC Genome Browser collection of RefSeq sequences (July 2003), 8433 exons were identified along with their neighboring introns (n = 13813) by sequence matching. Multiple occurrences of neighboring introns were removed. For the GO term analysis, we used a more stringent cutoff and only examined genes in the 95^th ^percentile according to their WKS-statistic (n = 431). GO term enrichment was determined using the Benjamini-Hochberg correction for controlling the false discovery rate at the .001 level.

To obtain a similar sized set of constitutive genes and exons so that the sequence comparisons would be balanced, we evaluated the lowest ~15^th ^percentile of genes according to the gene-level WKS-statistic and extracted the exons within these genes that scored below the median exon-level KS-statistic. Using the UCSC Genome Browser collection of RefSeq sequences, 8113 of these exons were identified along with their neighboring introns (n = 10081). Multiple occurrences of neighboring introns were removed.

### Motif analysis

We performed a search for over-represented kmers in our exonic and intronic sequences using the RESCUE-ESE method as described in [12]. Briefly, using the Perl programming language, counts for all kmers between two sets of sequences are tallied and evaluated for significant over-representation between the sets using a Z-test, and all kmers with Bonferroni-adjusted p-value < 0.01 were clustered and aligned into groups of motifs. Distances between motifs were defined as the absolute deviation between the nucleotide frequencies of two aligned positions in the best local alignment. Motif pairs with distance less than 2 were deemed as a motif match. For kmer calculations with exons, the comparisons are 1) exons versus introns and 2) constitutive versus alternative exons. We also considered two comparisons for each of 5' and 3' SS based on splice site strength scored by the position-specific weight matrices and applied the 1st and 3rd quartiles of all known splice sites as the cutoffs for weak and strong splice sites: 3) weak versus strong splice site exons and 4) strong versus weak splice site exons. For kmer calculations with introns, the comparisons are 1) introns versus exons and 2) flanking introns of constitutive versus alternative exons; 3) flanking introns of weak versus strong splice site exons and 4) flanking introns of strong versus weak splice site exons. All sequence analyses used intronic/exonic sequences up to 200 bp from the SS and excluded the splice site regions that cover the first 5 bp at each end of exons and the first 20 bp and 10 bp for the 3' and 5' ends of introns respectively.

In addition, we searched for novel sequence motifs utilizing the whole data set without the need to dichotomize exons into AE and CE by applying the correlation-based method REDUCE [50] to the splice site proximal sequences and our exon-level score for measuring exon skipping (n = 83789). REDUCE enumerates all possible 5–6 base pair kmers and finds those that show significant correlation with expression values from a single microarray experiment. Multiple kmers are identified by iteratively removing the significant kmers from sequences and re-evaluating the correlation between the remaining kmer frequencies and residuals of the linear regression fit from the previous run (*i.e.*, subtracting the contribution from the previously selected kmer(s)). We masked the splice sites by removing 5 bp at the 5' and 3' end of each exon. The output lists all significant kmers with p-values adjusted using the Bonferroni correction.

To look for motifs in the development-related AS exon sets, we examined the genes that had appropriate keyword hits (see below). Within these genes, sequences were extracted for exons predicted to be AE based on our exon-skipping score. De novo motif finders MEME [61] and BioProspector [62] were applied to these selected exon sequences subtracting the first 5 bp at either end of each exon. BioProspector was run with the following options: only examine the forward stand (-d), assume every sequence has a motif (-a 1) and motif width range from 6 to 18 (-w 6,8,10,12,14,16). MEME was run with the following options: default value of only looking at the forward strand, DNA sequence (-dna), several choices for the expected number of motif occurrences (zero or one in each sequence -zoops, one in each sequence -oops, variable number -tcm) and the motif width (-w 6,8,10,12, and the range -minw 6 -maxw 20). For each of the 15 MEME runs and the top three motifs from the 6 BioProspector runs, we aligned the consensus sequences for the final predicted motifs with ClustalW [74]. All motifs and alignments are displayed in the Figures using WebLogo [75]

### PubMed keyword search

For all genes in the Genome Browser RefSeq transcript list, the software R , with the "annotate" and "XML" packages was used to search each abstract of the references listed in the PubMed report for this gene. A hit for a gene was defined if the keyword(s) occurred in at least two of the abstracts for the gene. For each keyword, we performed a Fisher's exact test to evaluate whether the keyword appeared more frequently than expected by chance in our gene set compared to all genes. For the *splicing *keyword we searched for either "splicing" or "splice".

## Abbreviations

AS: alternative splice; AE: alternatively spliced exons; CE: constitutively spliced exons; CV: coefficient of variation; EJ: exon-junction; ESE: exonic splicing enhancers; KS: Kolmogorov-Smirnov; WS: Wilk-Shapiro; SS: splice site; ROC: receiver operating characteristic.

## Authors' contributions

KK and YHY performed the analysis, contributed to the design of the study and wrote the manuscript. RFY conceived the design of the study, coordinated the work and edited the manuscript. All authors read and approved the final manuscript.

## Supplementary Material

Additional file 1 **This file contains supporting tables:** Table S1: Significant GO terms for top scoring alternatively-spliced genes. Table S2: Motifs found in alternative exons within 200 bp of the 5'SS. Table S3: Motifs found in alternative exons within 200 bp of the 3'SS. Table S4: Motifs found in alternative exons within 200 bp of the 5'SS and 3'SS. (weak splice site strength comparison). Table S5: Motifs found in constitutive exons within 200 bp of the 5'SS. Table S6: Motifs found in constitutive exons within 200 bp of the 3'SS. Table S7: Motifs found within 200 bp of the 5'SS of 3' introns flanking alternative exons. Table S8: Motifs found within 200 bp of the 3'SS of 5' introns flanking alternative exons. Table S9: Motifs found within 200 bp of the 5'SS of 3' introns flanking constitutive exons. Table S10: Motifs found within 200 bp of the 3'SS of 5' introns flanking constitutive exons. Table S11: Number of significant kmers in predicted exons and flanking introns and randomly selected exons and flanking introns. Table S12: Significant GO terms for genes with development-associated alternative splicing in brain. Table S13: List of exons predicted to undergo development-related AS. Table S14: Binding sites for SR proteins. Click here for file

Additional file 2 **This file contains additional results and methods.** Click here for file

Additional file 3 **This file contains one supporting figure: **Figure S1: Differences in log-intensities (y-axis) between fetal and adult tissues for SR proteins listed in [20]. There are two replicates for each fetal and adult tissue, which are labeled with the same color. Tissues (brain, lung and liver) are labeled in different colors. Click here for file
