# Supplementary material for: Prediction of alternatively skipped exons and splicing enhancers from exon junction arrays
Source: BMC Genomics. 2008 Nov 20;9:551. doi: 10.1186/1471-2164-9-551 (PMC2631580; doi:10.1186/1471-2164-9-551)
Supplement: Additional file 2 — This file contains additional results and methods. [file 1471-2164-9-551-S2.doc]

**Supporting Text**

**Splice Site Comparison**

To correct for gene effects, for each exon we took the percentile rank of the splice site score relative to all splice site scores for the gene in which it occurs and then compared these percentiles between the AE and CE. Percentiles were smaller in the AE (Mann-Whitney test p-value = 6.5x10^-4^ for 3’SS and 2.0x10^-5^ for 5’SS) indicating that compared to CE, AE have weaker splice sites relative to other exons in their respective gene.

**Intronic splicing regulatory elements**

In addition to exonic splicing enhancers, there are also known intronic elements that positively or negatively affect splicing. The intronic suppressors are especially important in support of the hypothesis that splice site recognition occurs in a background of repression, implicating that compared to CE, sequences flanking AE might be enriched for suppressors, which may cause those exons to be skipped. We applied similar contrast analysis to identify intronic motifs associated with AE, by selecting for kmers over-represented in introns flanking AE versus CE-associated introns and in introns versus exons. These AE-associated motifs (Figure 4, Additional file 1, Tables S7 and S8) are either A/T-rich or resemble the 5’ splice site signal by containing the GT dinucleotide. Similar to the G/C-enrichment in CE, the A/T-enrichment in introns flanking AE may be explained by intron composition bias, since AE-flanking intronic sequences are also part of a much longer intron when the exon is skipped, and longer introns are known to have higher A/T content [1]. For this set of sequences, introns flanking alternative exons are longer with a median length of 1507 base pairs compared to those flanking constitutive exons with a median length of 1175 base pairs (Mann-Whitney test p-value =6.1x10^-34^). The median AT content in introns flanking alternative exons is ~.62 compared to ~.53 in introns flanking constitutive exons (Mann-Whitney test p-value << 10^-35^). The abundance of decoy splice sites in the proximity of real splice sites of AE also may explain why an AE is prone to be skipped, presumably by competitive base-pairing to the snRNA, and hence interfering with normal assembly of the spliceosome.

Selecting for kmers over-represented in introns flanking CE versus AE and in introns versus exons, we performed a similar analysis to identify CE-associated intronic features and recovered the pyrimidine (C/T) tract motif and known G-triplet motif which can be bound by the splicing factor hnRNP H [2, 3] (Figure 4, Additional file 1, Tables S9 and S10). For the 3’ end, this pyrimidine rich motif is slightly stronger than the 5’ pyrimidine rich motif which has higher A/G content. Although we removed 20bp from the 3’ end to avoid identifying the 3’SS and pyrimidine tract, the discovered pyrimidine-rich motif is likely to be part of a longer pyrimidine tract, which is required for efficient splicing and located between the branch site and the 3’SS [4].

**Randomization trials**

As a means of computational validation for the results obtained using RESCUE, we ran several randomization trials. Exons from the complete data set were randomly assigned as alternatively or constitutively spliced. This procedure was repeated five times and the resulting randomized exon sets were selected to be the same size as the AE and CE predicted from our method. To be consistent with our original prediction methods, we then selected the flanking introns of the randomized exon sets. Note however that the randomization intron sets may not be the exact size as our predicted AE and CE associated introns, but we do not expect this to affect the results.

The results of RESCUE on the randomization trials are reported in Additional file 1, Table S11, which lists the number of kmers identified for each random set, along with the number of kmers identified using the original set. In all comparisons, several hundred kmers were predicted in the AE and CE exons and associated introns, but for the randomization trials only 11 kmers on average were predicted.

**1. Hurst LD, Brunton CF, Smith NG: Small introns tend to occur in GC-rich regions in some but not all vertebrates. *Trends Genet* 1999, 15(11):437-439.**

**2. McCullough AJ, Berget SM: G triplets located throughout a class of small vertebrate introns enforce intron borders and regulate splice site selection. *Mol Cell Biol* 1997, 17(8):4562-4571.**

**3. Han K, Yeo G, An P, Burge CB, Grabowski PJ: A Combinatorial Code for Splicing Silencing: UAGG and GGGG Motifs. *PLoS Biology* 2005, 3(5):e158.**

**4. Hastings ML, Krainer AR: Pre-mRNA splicing in the new millennium. *Curr Opin Cell Biol* 2001, 13(3):302-309.**
