# Supplementary figures and images for: Prediction of alternatively skipped exons and splicing enhancers from exon junction arrays
Source: BMC Genomics. 2008 Nov 20;9:551. doi: 10.1186/1471-2164-9-551 (PMC2631580; doi:10.1186/1471-2164-9-551)

**Figure S1:**


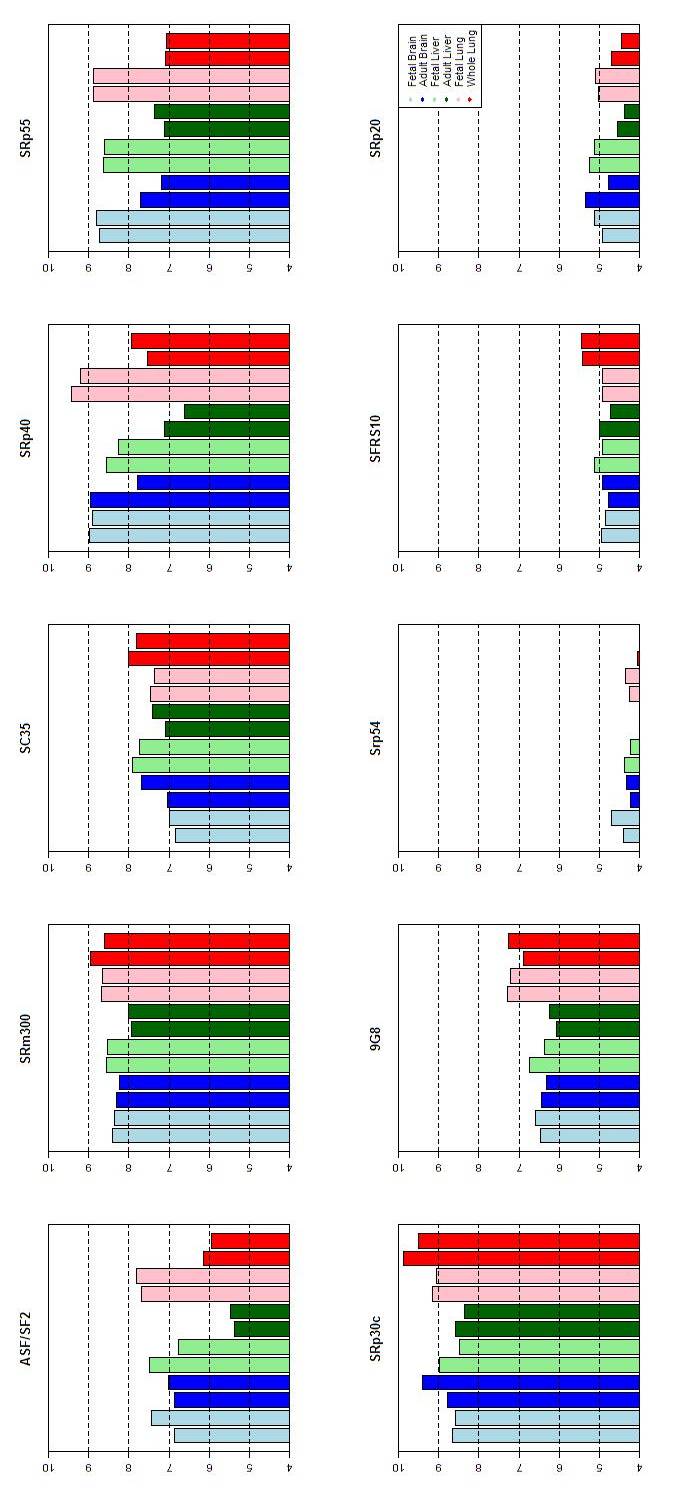

Supplement: Additional file 3 — This file contains one supporting figure: Figure S1: Differences in log-intensities (y-axis) between fetal and adult tissues for SR proteins listed in [20]. There are two replicates for each fetal and adult tissue, which are labeled with the same color. Tissues (brain, lung and liver) are labeled in different colors. [file 1471-2164-9-551-S3.doc]
